# Observations on the Tread-Mill

**Published:** 1823-11

**Authors:** B. Hutchinson

**Affiliations:** Fellow of the Royal College of Surgeons in London.


ART. III.—*Observations on the Tread-Mill.* By B. HUTCHINSON, Esq. Fellow of the Royal College of Surgeons in London.

IT must be allowed that the public (equally with ourselves, who are bearing the very easy weight of a very amicable controversy,) are much indebted to you for the admission into your Journal of observations, which, on a first and superficial view of the subject, might appear to be foreign to the general and professed objects of your useful and excellently-conducted work. When, however, we take into our consideration the improvement of the health of the numerous inhabitants of our prisons, both physical and moral; and that the question of the tread-mill's expediency or inutility is deeply involved in this inquiry; all sentiments of that nature must completely give way to the gradual establishment of truth and positive facts, and to the exclusion of all hypotheses and preconceived theories.

I beg thus publicly to thank Dr. Good for his donation of Sir John Cox Hippisley's "Correspondence on Prison Discipline," and for the candour and liberality with which he has received my remarks. The careful perusal of this "Correspondence" has not produced the slightest change in my sentiments on the subject, which has so seriously and laudably engaged his closest attention. My personal and daily observations of the excellent and efficient operation of the tread-mill, as far as regards the health of the prisoners in the house of correction at Southwell, tend to impress on my mind a conviction of the accuracy and truth of my statements in the communication which you did me the honour of publishing two months back, and which will be further illustrated in the course of this letter by an experimental inquiry, made before the Rev. J. T. Becher, one of the visiting justices of this house of correction.

Although I cannot attach that importance to my omission of dates and numbers, which Dr. Good considers as points of the utmost moment in the present inquiry, I shall nevertheless undertake to furnish the Doctor with the information he solicits, and which I have obtained through the permission of Mr. Becher. This information cannot be conveyed in a more distinct manner than by detailing the questions which were this day asked Mr. Mole, the active and intelligent governor of the prison, by Mr. Becher, and the replies which he made to them.

1. When was the tread-mill established?—December 23, 1822.

2. What number of men have been employed on an average?—Daily average, from December 23, 1822, to 17th September, 1823, both days inclusive, $44\frac{183}{232}$.

3. What number of men are this day employed?—Fifty.

4. What complaints have been made to you by any prisoner employed in this mode of discipline?—None; not a single complaint.

5. What accidents have happened to the men, and what to the machinery?—One man had his foot slightly bruised, but this occurred through his own wilful neglect in coming off the wheel. No accident has happened to the machinery.

6. At what periods are the men relieved?—One man gets off the wheel every minute, allowing each never less than one-fourth rest, and occasionally nearly one-half, according to the number employed at each wheel: viz.—If twelve are employed at a wheel, nine are on the wheel, and three off, which gives one-fourth rest. If ten are employed at a wheel, seven are on the wheel, and three off, which gives near one-half rest; one man getting off, and one on, every minute.

7. In what periods do the wheels revolve?—Two of the wheels on the ground-floor make two revolutions in a minute; and the two wheels on the upper floor make three revolutions while those on the ground-floor make four.

8. What is the rise of the steps?—Eight inches.

The dietary of the house of correction at Southwell cannot be considered as one of the highest class; but from which the prisoners on the wheel have received a sufficiency of nutriment to preserve the functions of the human machine in a state of healthful vigour. I have not found any necessity to increase the quantity or quality of the allowance, the dietary remaining the same as before the tread-wheel was established in this prison. This dietary consists of a loaf of coarse wheaten bread, weighing one pound and three quarters, and a pint of new milk to breakfast; one pint of oatmeal-gruel at noon, and the same in the evening; and a quarter of an ounce of salt per day. Awakened as the public mind has been to the subject of prison dietaries, by the parliamentary investigation which has lately taken place respecting the Millbank Penitentiary, I may perhaps be permitted, after an experience of twenty-eight years, to make some professional remarks upon this subject. I find it stated in the "Rules for the Government of Gaols," published by the Society for the Improvement of Prison Discipline:—"N. B. One pound and a half of good wheaten bread, and a quart of gruel or soup daily, or a ration equivalent to this, is considered quite sufficient for the maintenance of a prisoner employed in ordinary labour," (p. 41;) and Mr. Webbe, surgeon of the house of correction in Cold-bath Fields, affirms that half a pound of solid flesh every other day, with good animal soup in the intermediate day, besides a sufficiency of bread and other farinaceous food, is necessary; as, without this increased diet, the workers on the tread-wheel would be soon in the situation of the convicts at the Millbank Penitentiary. My practical knowledge is at direct variance with both these dietaries; for I pronounce our present dietary sufficient for any employment that can be exercised within the walls of a prison, having adequately sustained the prisoners upon it while they were excavating the foundations of the tread-mill in a treacherous soil, and digging and wheeling from a depth fifteen feet beneath the surface of the ground, as well as in other laborious occupations. Collecting my information from other gaols, when originally directed to determine the dietary, I proposed daily one pound and three-quarters of coarse wheaten bread, half a pint of gruel at breakfast, and one pound of boiled potatoes to dinner. Not being satisfied with the general state of health, I recommended the addition of half a pint of gruel before retiring to rest. Finding an improvement in the scale of health, though not completely corresponding with my wishes, and apprehending that a diet strictly farinaceous, like that then in use, would admit of considerable improvement by introducing a substance partaking in some measure of the properties of animal food, I advised the substitution of one pint of milk daily instead of one pound of potatoes; and it is gratifying to announce that the result has been completely satisfactory, and that I am enabled to state my conviction of its sufficiency both for the purposes of labour and of general health,—not as a theoretical surmise, but as an indisputable fact falling within my knowledge, in discharging the duties of my official situation. As surgeon of the establishment, the visiting justices allow me a discretionary power of improving the scale of diet according to the exigencies of particular cases; which power I, of course, find an almost daily occasion to exercise among the patients sent to the infirmary: it has never occurred, however, in a single instance, that an improved diet has been rendered necessary from any excessive fatigue, or from any morbid derangement produced by the exercise of the tread-wheel.

In opposition to the assertion that the labour and mischiefs of this machine exceed that of any invention which has hitherto undergone the test of experience, Mr. Mole, the governor, on questioning a prisoner in the presence of Mr. Becher, whether he would prefer breaking flax with the patent machine to the labour of the tread-wheel, gave a decided preference to the wheel, in consequence of the labour of the latter being so much less fatiguing than that of the former. Dr. Good's question respecting the employment of female prisoners on the tread-wheel at Southwell, is answered in the negative; female delinquents never having been ordered, at present, by the visiting justices, to be subjected to this punishment.

In reference now to Sir J. Cox Hippisley's correspondence, pages 11, 12, 13, and 14, I beg to state that his former objections to the use of the tread-wheel, (which I shall briefly recapitulate,) are completely and in the most satisfactory manner contradicted by the personal communications of between twenty and thirty prisoners, who had been labouring for different periods at the tread-wheel. These objections consisted "in the treading on tiptoe up an endless hill with the body bent forward, and with the hands rigidly and unremittingly grasping a rail for support; that, in consequence hereof, a most distressing thirst, debilitating perspiration, and *actual loss of flesh,* are often produced. That not only severe exhaustion, but strains upon the organs and muscles immediately called into exercise, in many cases highly injurious to health, have actually taken place on various occasions, and, in the opinion of a large body of physicians and surgeons of the highest rank and respectability, who have minutely examined into the subject, are necessarily threatened at all times. That the concurrent testimony of numerous medical practitioners, of high character and extensive experience, has proved that habitual labour of a like description, as that of mariners, and even of a lighter kind, as the ladder treading in thatching, and among masons, labourers, miners, &c. has a gradual tendency to produce ruptures and varicose veins, or nodulous tumors on the legs; and in numerous instances has produced them. Whence it has been reasonably apprehended by other practitioners, of great talents and attainments, who have particularly attended to this machine and its effects, that a stated and longer employment upon it than has hitherto been experimented in any prison, in consequence of its being of novel introduction, will necessarily give a still greater tendency to the same injuries; and, in the end, more certainly and more extensively induce them among those who are sentenced to its morbid discipline. That, for these and similar reasons, the unhappy culprits have a horror of the mill, and would sooner undergo, as they all declare, any fatigue, or suffer any deprivation, than return to the house of correction, when once released."

In Dr. Good's letter to Sir J. Cox Hippisley, page 26 and 27 of the same volume of "Correspondence and Communications," are the following observations:

"From the tortuous attitude and uneasy motion manifestly displayed in mounting the endless hill of this mighty cylinder, upon the toes alone, with the hands fixed rigidly on the horizontal bar, and the body bent forward to lay hold of it, I could not but conclude, not only that the prisoner is hereby deprived of all the healthful advantage of athletic exercise, but must be fatigued from the outset, and perpetually in danger of the cramp, breaking the Achilles tendon, and forming aneurismal and varicose swellings of the legs; and that, if females were to be worked at the wheel, the same common cause of irksome and distressing exertion operating on the loins and many of the abdominal muscles, must, of necessity, in various instances, accelerate the period of menstruation; and, even where it does not force it forward before its proper time, render it excessive, and lay a foundation for many of the most serious chronic maladies with which the female structure can be afflicted. In the Cold-bath Fields' prison, I found, upon close inquiry, that the prisoners frequently complained of stiffness and numbness in their hands, of pains in their loins and their legs; and that they were thrown into a profuse perspiration, and so completely exhausted in the course of a single round, or quarter of an hour's task-work, as to induce them to drink very largely of cold water as soon as the fifteen minutes were completed, although it is calculated that this up-hill exercise does not exceed the average of two miles in six hours, and consequently does not amount to half a quarter of a mile in the course of the fifteen minutes to which the task-time extends; evidently proving that it is the *nature* of the labour, its quality, and not its quantity, that occasions such violent effects, and constitutes the terror with which the tread-wheel is contemplated. I do not know that any of these maladies, which, from the recent use of the wheel, could not be of long standing, had produced any ill effects upon the constitution of the prisoners, or permanently undermined their health."

In page 32 of the same volume, Dr. Good describes his visit to, and his observations on the operation of the tread-wheel, at the Cold-bath Fields prison; of which I must beg leave to present a brief copy.

"I inspected the men as they descended in rotation from the wheel, at the end of the quarter of an hour's task-work, and made room for fresh relays. Every one of them was perspiring, some in a dripping sweat. On asking them separately, and at a distance from each other, where was the chief stress of labour, they stated in succession, and without the least variation, that they suffered great pain in the calf of the leg, and in the ham; whilst most of them, though not all, complained of distress also in the instep. On examining the bottom of their shoes, it was manifest that the line of tread had not extended farther than from the extremity of the toes to about one-third of the bottom of the foot; for in several instances the shoes were new, and between this line and the heel altogether unsoiled: a fact, however, that was as obvious from the position of the foot while at work, as from the appearance of the shoe at rest. Several of the workers seemed to aim at supporting their weight by bringing the heel into action, the feet being twisted outwards; and, on inquiring why this was not oftener accomplished, the reply was, that, though they could gain a little in this way, it was with so painful a stress of the knees, that they could only try at it occasionally. The palms of their hands, in consequence of holding tight to the rail, were in every instance hardened, in many horny, in some blistered and discharging water. The keeper, who accompanied us, admitted the truth of all these statements, and added, that it was the ordinary result of the labour! and that use did not seem to render it less severe: for those who had been confined long appeared to suffer nearly, or altogether, as much as those who were new to the work; thus confirming a remark I long since took the liberty of making to you: I mean that, when an organ is directed to any kind of labour for which it is not naturally intended, no perseverance will ever give it facility of action, or take off the original distress."

I have taken the liberty of making these copious extracts from Sir J. Cox Hippisley's volume, under the impression that the book is not in the hands of the majority of the readers of your Journal, and who would be unable to form a proper or just estimate of the arguments on each side of the subject, without some assistance of this description. My labour of refutation will be rendered very easy and compressed, by transcribing, in the first place, a letter published in the "John Bull" Newspaper, of September 14th, from Mr. Jackson, the very intelligent surgeon of the Guildford house of correction; followed by the result of an examination of between twenty and thirty of the prisoners in the Southwell house of correction, just taken from the wheel.

"TO JOHN BULL.

"Sir,—I read, with no small degree of surprise, in your paper of the 24th of August, some observations relative to the use of the tread-mill, together with copious extracts from Sir J. Cox Hippisley's work, in which a truly horrible account is given of the sufferings of the prisoners in the house of correction at Cold-bath Fields. These observations and extracts are so completely at variance with the state of things as they exist in the house of correction at this place, (Guildford,) more especially with regard to the effects of the wheel on the female prisoners, that I am induced to trouble you with an account of the weight of all the women who have been admitted into this prison between the 1st of May and the 1st of September; by which statement I trust I shall be able to show that, if increase of weight be a proof of improved health, the women are in better case than when they were admitted."

[Here follows a statement of seventeen female prisoners, all of whom, with the exception of three, had gained, in the course of four months, several pounds in weight.]

"By this statement you will perceive that, with very few exceptions, the women have gained considerably in weight; and, of those who have lost weight, two were labouring under disease at the time of admission, on which account they were seldom put upon the wheel. I beg leave to add, that I have witnessed some of the bad effects of the wheel on the hands of the female prisoners, as mentioned in Sir J. Cox Hippisley's work: and, in regard to the dreadful consequences described by Dr. Mason Good, as *likely to result* from the labour of females on the tread-mill, I most positively declare that no such consequences have existed here. The prisoners, and more especially the women, are in good health. The few cases of sickness which have come under my care, have generally been chronic cases of long standing, and were so at the time of admission into this place; and I do most solemnly declare that I have, as yet, witnessed no bad effects on the legs, arms, or bodies, of the prisoners from the use of the tread-mill.

"My object in troubling you with this is to elicit the truth, and to do away what appears to me, judging from its effects in this place, a groundless objection to a humane, useful, and harmless instrument of punishment. If the above be deemed worthy of a place in your paper, you will oblige me by inserting it. I am, Sir, your obedient servant,

"EDWARD JACKSON,

"*Surgeon to the House of Correction at Guildford.*"

"*September 2d,* 1823."

"P.S.—On visiting the prison this day, I found that the mill was not at work, on account of the want of corn: the men were in their different airing wards; and the women all employed in needle-work, at which they appeared to be very expert, not one of them complaining of horny or blistered hands."

The same Number of "John Bull" contains a letter addressed to the "Editor of the London Medical and Physical Journal," from Sir J. Cox Hippisley, with some remarks on my former communication to you on the subject of the tread-wheel. Sir John appears to retain his former sentiments and antipathies, without, however, producing any new arguments in any way bearing upon the point in question, or in the slightest degree weakening the force of facts so formidably arrayed in opposition to delusive hypothesis and vague conjecture.

I shall now beg leave to state the result of the examination before alluded to at the Southwell house of correction, in full confirmation of the opinions I have so frequently offered. In this examination, I was most kindly and ably assisted by the Rev. John Thomas Becher, one of the visiting justices of this prison, and in the presence of Mr. Mole, the governor. The prisoners were distinctly informed that this examination would operate neither to their advantage nor detriment.

| *Names.* | *Number of Months or Weeks on the Wheel.* | *General Observations.* |
|---|---|---|
| Wm. Paton, | 10 months, | His knees, legs, and thighs, fatigued in an evening. No swellings in his legs, nor cramps. Walks with the greatest ease when fronting the wheel. The labour not greater than that of a common day-labourer. |
| William Allen | 5 months, | No pains nor swellings of any kind. Could step laterally on the wheel the whole day, but eases himself by changing his position. |
| J. Staniland, | 9 months, | Can walk laterally the whole day; or either in front or to the back of the wheel. No pains of any sort. |
| G. Cullen.... | 9 months, | Treads in front with the greatest ease, but often changes his position; can walk backwards. Has no pains nor swellings. |
| W. Clewes.. | 9 months, | Walks fronting the wheel the easiest; can also walk sideways and backward. |
| J. Abbott .. | 5 months, | Treads in front with the greatest ease. No pains. |

| Names. | Number of Months or Weeks on the Wheel. | General Observations. |
| --- | --- | --- |
| J. Hall, .... | 5 months, | Treads easiest laterally, and could tread the wheel in that position the whole day. |
| W. Calton,.. | 3 months, | Treads laterally, backwards and forwards. Complains of no pains, but of weariness in the evening. Sleeps well. |
| J. Kirk, .... | 5 months, | Treads laterally the easiest. No pains in his knees, nor weariness at night. |
| J. Northway, | 3 weeks, | Treads fronting the wheel. No pains, but wearied in an evening. |
| W. Simpson, | 6 weeks, | Treads laterally with the most ease. No pains. |
| T. Wheely,.. | 6 weeks, | Laterally the whole day. Has been a soldier, and experiences the same fatigue as produced by a walking drill, but not so severe as a balance step. The exercise not so severe after he had been used to it. No pains. |
| Thomas Northwainer, | 6 weeks, | Agrees with the above. Compares it to the balance step, and the pains go off after rest. No pains. |
| J. Slack,.... | 6 weeks, | Treads the easiest in a lateral direction; can also tread backwards. No pains. |
| W. Hatter,.. | 6 weeks, | Does not think the exercise of the tread-wheel so severe as that of the walking drill. Treads the easiest on the front, and has no pains nor swellings. |
| Wm. Torr, .. | 6 weeks, | Treads laterally the easiest. Labour somewhat like the balance step, but more fatiguing. A feeble man. |
| R. Burns, .. | 6 months, | Treads easiest by changing his position. Not so laborious an employment as thrashing in a barn. No pains, swellings, nor much fatigue. |
| W. Wigley, . | 3 months, | Treads fronting the wheel more easily than laterally, but can do either. The labour not so severe as getting iron-stone. No pains. |
| J. Bliton, .. | 3 months, | Treads laterally, in front, or in any direction, but prefers facing the wheel. No complaints of any kind, excepting fatigue in the evening. |
| J. Wilkinson, | 4 months, | The lateral tread is preferred. Without pain or complaint. |
| J. Orridge,.. | 5 months, | Treads easiest facing the wheel, but has an advantage in a change of position. No complaints of pain nor of swelling, but of great fatigue in an evening. |
| W. Omfield, | 6 months, | The lateral position the easiest, and says that the labour is about as severe as that of sawing. He has varicose veins in his legs; but had them previous to labouring on the wheel, or being committed to this prison. |
| J. Hallam, .. | 11 months, | The lateral position the easiest, and has no swellings nor pains. |
| T. Cliff, .... | 11 months, | Can tread in almost any direction. Complains of fatigue from the exercise; but has suffered neither permanent pains, swellings, nor any deprivation of rest or appetite. |

However diffident I may feel respecting my own judgment, I stand on this occasion supported by the concurrent opinion of those accustomed to minute and accurate investigation. After the twenty-four prisoners, whose depositions are here given, had been individually examined by Mr. Becher, without any previous knowledge of the purpose for which they were brought before him, and had, by their unanimous testimony, produced a confirmation of my original statement, twenty-six fellow prisoners, who were also employed upon the tread-wheel, were summoned; and, the whole fifty being collected, were desired to state, without intimidation, whether they experienced any disorder, or any other sensations than those which are common to the soldier at his drill, the mechanic at his shop, or the labourer in his barn? The result was, that every man, exercising what is denominated a laborious occupation, had frequently sustained, from a continuance, much severer and more distressing employment; that the sensations and pains were nearly allied to those succeeding a drill, especially at the balance step; and that the frame-work knitters and lace manufacturers of this county, who exercise an art requiring a peculiar flexibility of muscle and an extreme delicacy of touch, were not in the slightest degree prevented from resuming their usual employments after their discharge from the tread-mill.

Mr. Mole, the governor, I may take the liberty of mentioning, is a man of indefatigable vigilance and acknowledged humanity: his whole time is devoted to the duties of his office; the prisoners, while on the tread-wheel, are always immediately before his window. He was originally bred at Birmingham to the manufacture of arms; after which he served as a lieutenant in the 32d Regiment for several years, with very considerable reputation. On leaving the army, he resumed the superintendance of a large manufactory at Birmingham; and I leave it for the public to determine whether such a person is likely to be deceived in matters submitted to his daily and hourly inspection; and whether his duty to the magistrates, as well as his own sentiments of justice and humanity, would not have impelled him long since to have made a formal representation, if all or any of the evils ascribed to this machine had existed within our prisons? With less circumspection in conducting the examination of the prisoners, it is neither impossible nor improbable that the hopes of reverting to that idleness which has disgraced our prisons, and drawn such multitudes within their walls, might have proved a powerful inducement to attempt delusion by representation arising rather from interested motives than from actual suffering.

The question of the utility or of the injurious tendency of the tread-wheel being, in my opinion, satisfactorily determined by the testimonies offered in the preceding pages, as well as by the concurrent opinions in its favour of the respectable medical gentlemen, whose sentiments on this subject have been publicly and most impartially given, I shall beg permission to state the enviable degree of health which has been obtained at the Southwell house of correction, by the excellent system of ventilation, cleanliness, and general good management, adopted and enforced by the visiting justices of that prison. I will first mention that one death only has occurred during the last five years,—Robert Smith, who entered the house of correction with symptoms of confirmed phthisis pulmonalis. So far, indeed, had structural disorganization advanced on his commitment, that he lived only a few days after his admission. The gross number of prisoners committed during that period (five years,) amounted to 3227; and the average number of prisoners daily confined for the last year, commencing 25th of June, 1822, and ending 24th of June, 1823, both of the above dates inclusive, amounted to $81\frac{238}{365}$. The number on the sick-list at this time is fourteen: many of these cases are of minor importance, and not one of them depending either on the effects of the tread-wheel or of incarceration.

As to the abandonment of the tread-wheel by Parliament, in the recent Gaol Act, I can only affirm that, among the several Prison Bills which during the last few years have been submitted to my inspection, I have not discovered that any such clause was ever adopted; neither am I acquainted with any provisions in any statute prescribing imperatively the particular mode of hard labour. The magistrates exercise their office gratuitously in favour, and to the great obligation, of the public: it is, therefore, I conceive, more consonant with the respect due to their office, that Parliament should promulgate the *general* system to be adopted in prisons for the punishment and reformation of criminals, and that the *details* regulating the mode of carrying the intentions of the legislature into effect should be governed by the judgment of the magistracy, according to local opportunities and existing emergencies.

*Southwell; October 1st, 1823.*

